# *Fusobacterium nucleatum* infection correlates with two types of microsatellite alterations in colorectal cancer and triggers DNA damage

**DOI:** 10.1186/s13099-020-00384-3

**Published:** 2020-09-29

**Authors:** Yoshiki Okita, Minoru Koi, Koki Takeda, Ryan Ross, Bhramar Mukherjee, Erika Koeppe, Elena M. Stoffel, Joseph A. Galanko, Amber N. McCoy, Temitope O. Keku, Yoshinaga Okugawa, Takahito Kitajima, Yuji Toiyama, Eric Martens, John M. Carethers

**Affiliations:** 1 Division of Gastroenterology and Hepatology, Department of Internal Medicine, University of Michigan, Ann Arbor, MI USA; 2 Department of Biostatistics School of Public Health, University of Michigan, Ann Arbor, MI USA; 3 Division of Gastroenterology and Hepatology, Departments of Medicine & Nutrition, University of North Carolina at Chapel Hill, Chapel Hill, NC USA; 4 Department of Gastrointestinal and Pediatric Surgery, Graduate School of Medicine, Mie University, Mie, Japan; 5 Department of Microbiology and Immunology, University of Michigan, Ann Arbor, MI USA; 6 Department of Human Genetics and Rogel Cancer Center, University of Michigan, Ann Arbor, MI USA

**Keywords:** *Fusobacterium nucleatum*, Colorectal cancer, Microsatellite instability, *CpG* island methylator phenotype, MSI-L, EMAST, MSH3, Mismatch repair, Inflammation

## Abstract

*Fusobacterium nucleatum* (*Fn*) is frequently found in colorectal cancers (CRCs). High loads of *Fn* DNA are detected in CRC tissues with microsatellite instability-high (MSI-H), or with the *CpG* island hypermethylation phenotype (CIMP). *Fn* infection is also associated with the inflammatory tumor microenvironment of CRC. A subtype of CRC exhibits inflammation-associated microsatellite alterations (IAMA), which are characterized by microsatellite instability-low (MSI-L) and/or an elevated level of microsatellite alterations at selected tetra-nucleotide repeats (EMAST). Here we describe two independent CRC cohorts in which heavy or moderate loads of *Fn* DNA are associated with MSI-H and L/E CRC respectively. We also show evidence that *Fn* produces factors that induce γ-H2AX, a hallmark of DNA double strand breaks (DSBs), in the infected cells.

## Main text

*Fn* is a common resident in the human gut mucosa and is an anaerobic bacterium that colonizes CRC tumors more frequently than adjacent normal mucosa. To date, most epidemiological studies using 16s rRNA sequencing or metagenomic sequencing methods have detected an increased level of *Fn* DNA and/or RNA in colorectal adenoma/carcinoma tissues or stools from tumor bearing patients, as compared with normal controls [1]. Furthermore, *Fn* infection is associated with specific subtypes of CRC that exhibits CIMP or MSI-H [2, 3]. These observations might suggest that *Fn* infection may contribute to a serrated pathway of CRC development [4]. On the other hand, tumor tissue infected with *Fn* exhibits an inflamed tumor microenvironment, rich in inflammatory factors such as IL6 or reactive oxygen species [5], leading to the assumption that *Fn* infection might also contribute to the generation of IAMA or L/E positive CRC [6–8]. Despite a strong association between *Fn* infection and colorectal cancer, there has been no evidence of *Fn* infection damaging the DNA of colon tissues. In this study, we show evidence that a degree of *Fn* infection may determine molecular characteristics of CRC, and that *Fn* infection may be carcinogenic.

**Fig. 1:**
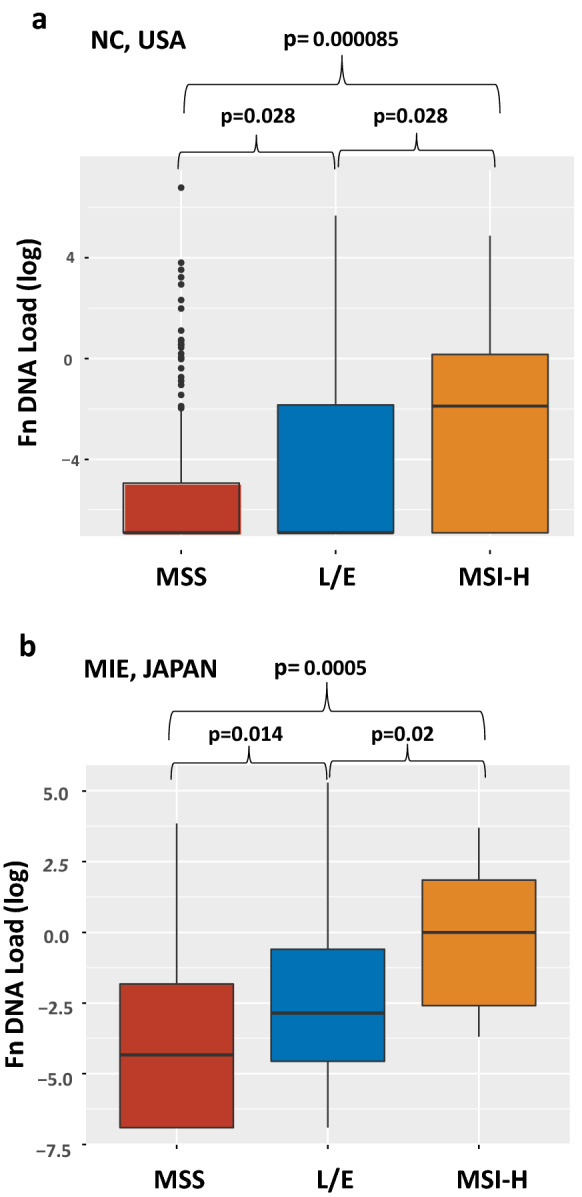
*Fn* DNA load in MSS, L/E and MSI-H CRCs. (**a**) Discovery cohort from North Carolina, USA. (**b**) Validation cohort from Mie, Japan. Absolute *Fn* DNA weight was normalized by the absolute tumor DNA weight present in the same DNA sample. Normalized values were converted to logarithmic scales. The data is depicted in the boxplot. Brown, blue and orange boxes represent the distribution of *Fn* DNA in MSS, L/E and MSI-H CRC, respectively. The thick horizontal line within in each box represents the median *Fn* DNA load. Dots in MSS column in the USA cohort represents outliers. The statistical difference in *Fn* DNA loads among MSS, L/E and MSI-H was tested using the Wilcoxon rank sum test. A p-value that is less than 0.05 is considered significant

**Fig. 2:**
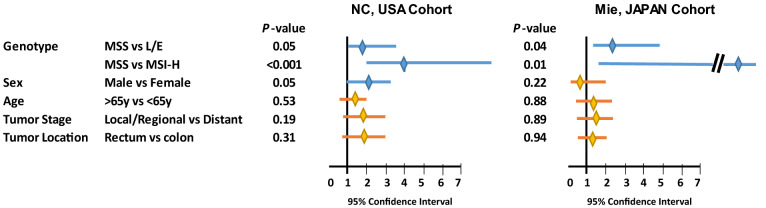
Logistic regression model for association between *Fn* infection and CRC molecular subtypes. Discovery cohort from North Carolina, USA (*left*) and validation cohort from Mie, Japan (*right*). Association was modeled by comparing MSS versus L/E or MSI-H. Positive for *Fn* infection was designated when the sample gave detectable *Fn*-specific PCR products by qPCR. The association was adjusted by sex, age, tumor location and tumor stage. The X-axis represents a range of 95% confidence interval (CI). Each horizontal bar indicates 95% CI for that variable. A *blue* or *orange bar* represents the significance or insignificance, respectively, between each variable and *Fn* infection. The *middle diamond shape* represents the value for the odds ratio. The *P*-values are shown after each variable

A total of 304 cases of unselected sporadic CRC from North Carolina [9, 10] were analyzed for MSI-H, MSI-L and EMAST [11, 12]. The amount of *Fn* DNA per nanogram of tumor tissue DNA was also determined by qPCR (see Additional file 1: Additional Materials and Methods). Thirty-eight cases (12.5%), 129 cases (42.4%) and 137 cases (45.1%) exhibited MSI-H, L/E and MSS, respectively. *Fn* DNA was detected in 116 of 304 (38%) CRC tumor tissues, ranging from 0.002 to 880 pg/ng of tissue DNA. When the quantity of *Fn* DNA was compared among MSI-H, L/E and MSS CRC, the *Fn* DNA load in MSI-H was the highest (MSI-H > L/E, p = 0.028; MSI-H > MSS, p = 0.000085) and the *Fn* DNA load in L/E was higher than in MSS (L/E > MSS, p = 0.028) (Fig. 1a). We then determined whether *Fn* infection was associated with MSI-H and/or L/E compared to MSS using a logistic regression model. In univariate analysis, *Fn* infection was associated with MSI-H at an odds ratio (OR) of 4.21 (p < 0.001) and was also associated with L/E at an OR of 1.74 (p = 0.03). When adjusted for sex, age, tumor location and tumor stage, MSI-H (OR = 3.99, 95%CI 1.85–8.9, p < 0.001) and L/E (OR = 1.68, 95% CI 1.00–2.84, p = 0.05) were independently associated with *Fn* infection (Fig. 2). To validate the above results, we analyzed 174 cases of CRC from Mie, Japan. Thirteen (7.4%), 69, (39.7%) and 92 cases (52.9%) exhibited MSI-H, L/E and MSS, respectively. *Fn* DNA was detected in 131 of 174 (75%) tumors, ranging from 0.0003 to 200 pg/ug tissue DNA. The quantity of *Fn* DNA was highest in MSH-H compared to L/E (p = 0.02) or MSS (p = 0.0005), and the *Fn* load was higher in L/E than MSS (p = 0.015) (Fig. 1b). *Fn* infection was associated with MSI-H at OR = 13.83 (p = 0.007) and with L/E at OR = 2.35 (p = 0.02) in univariate logistic regression analysis. Multivariate analysis adjusted by sex, age. tumor location and stage showed that MSI-H (OR = 13.67, 95% CI 1.63–1789.13, p = 0.01) and L/E (OR = 2.23, 95% CI 1.05–4.95, p = 0.04) were significantly associated with *Fn* infection compared to MSS (Fig. 2).

**Fig. 3:**
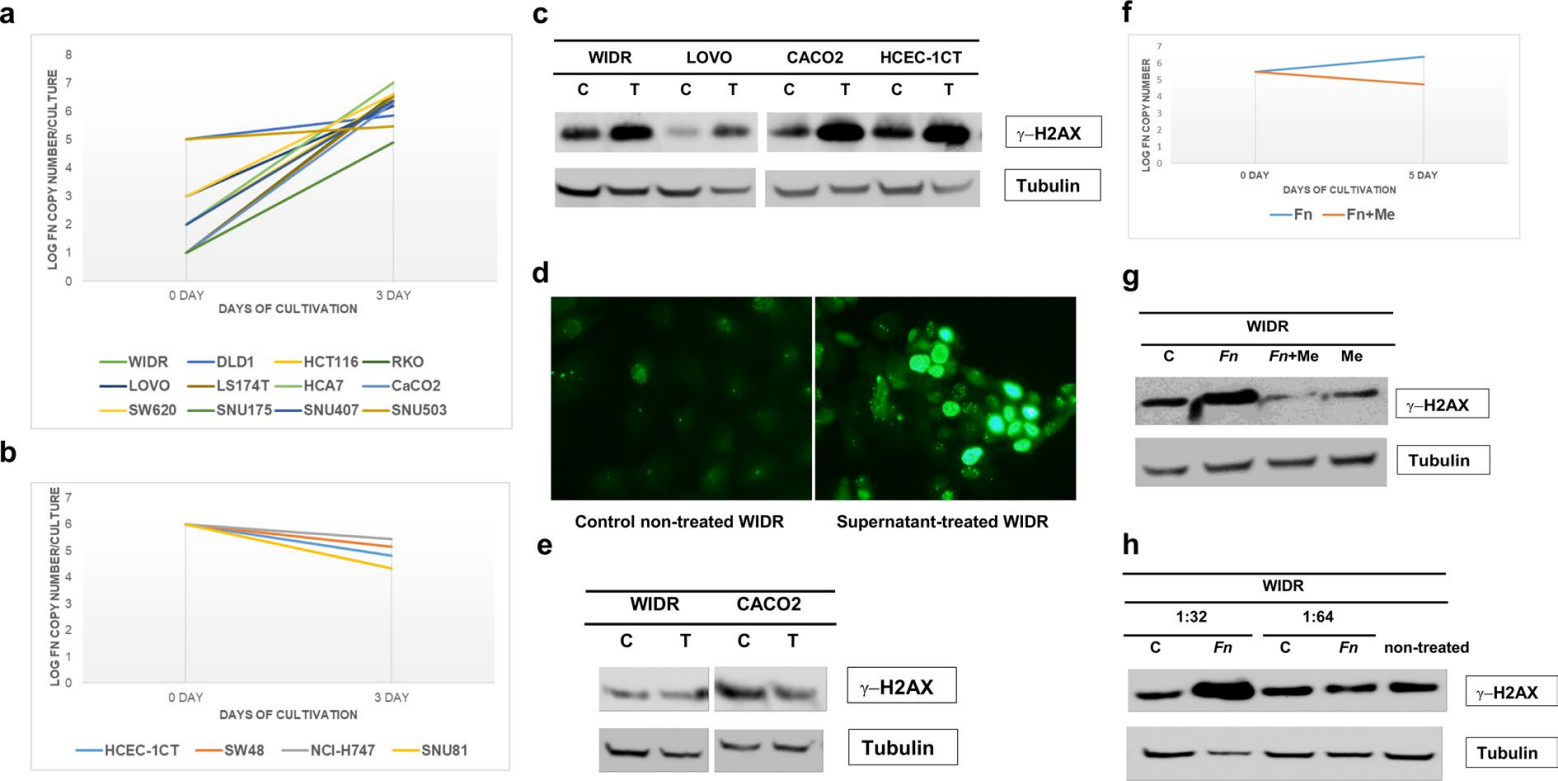
Aerobic growth of *Fn* and induction of γ-H2AX by *Fn.* (**a**) Twelve colon cancer cell lines (WIDR, DLD1, HCT116, RKO, LOVO, LS174T, HCA7, CaCO_2_, SW620, SNU175, SNU407, and SNU503) were infected with *Fn* strain EAVG_002. During a 3-day incubation, an increase in *Fn* copy number was observed in co-cultures with these cell lines. (**b**) No increase in *Fn* copy number was observed in co-cultures with 4 different colon cancer cell lines (HCEC-1CT, SW48, NCI-H747 and SNU81). *X-axis*: days of cultivation; *Y-axis*: log of *Fn* copy number per culture. (**c**) WIDR cells were infected with *Fn* EAVG_002 strain at a multiplicity of infection of 1 under 5%CO_2_/21%O_2_ for 1 week. Supernatants were collected, centrifuged, and filtered through a 0.2 μm porous membrane. 2 × 10^5^ cells (WIDR, LOVO, CaCO_2_ and HCEC-1CT) were exposed to the supernatants for 9 hr. Cell lysates were analyzed for induction of γ-H2AX by Western blotting using anti-γ-H2AX mouse monoclonal antibodies. C: control non-treated; T: supernatant treated. Treated cells expressed more γ-H2AX than control non-treated cells. (**d**) Immunofluorescent staining of *Fn* supernatant-treated WIDR cells with anti-γ-H2AX mouse monoclonal antibodies. Brighter nuclear γ-H2AX signal is evident in treated cells. (**e**) Supernatant from HCEC-1CT cell culture infected with *Fn* EAVG_002 strain at an MOI of 1 under 5%CO_2_/21%O_2_ conditions failed to induce γ-H2AX in supernatant-treated WIDR and CaCO_2_ cells. C: control non-treated; T: supernatant treated. There was no difference in the amount of γ-H2AX between control non-treated and supernatant treated cells. (**f**) Treatment of co-culture between WIDR and *Fn* with metronidazole inhibited *Fn* aerobic growth (red line). Blue line represents growth of *Fn* without metronidazole. (**g**) Metronidazole abolished γ-H2AX induction by *Fn*. Supernatants were collected from 4 cultures (C: control non-treated cells; Fn: *Fn* infected cells; Fn + Me: *Fn* infected cells treated with metronidazole; Me: non-infected cells treated with metronidazole alone) and tested for induction of γ-H2AX in WIDR cells. (**h**) Bacterial medium in which *Fn* grew anaerobically was tested for γ-H2AX induction. The *Fn* grown medium and control fresh bacterial medium was diluted by Dulbecco's modified Eagle medium with 10% fetal bovine serum at 1:32 and 1:64 ratio and then exposed to WIDR cells. C: WIDR cells were treated with a diluted fresh bacterial medium; Fn: WIDR cells were treated with diluted *Fn* grown medium. Induction of γ-H2AX was detected at 1:32 but not at 1:64 dilutions of *Fn* grown medium

To explore whether *Fn* infection causes cellular DNA damage (see Additional file 1: Additional Materials and Methods), we first determined the ability of human colon cancer cells to support infection. When each of 16 human colon cancer cell lines was co-cultured with *Fn* in 5% CO_2_/21% O_2_ conditions, *Fn* grew aerobically in 12 of 16 cell lines (Fig. 3a), but not in 4 cell lines (Fig. 3b). Furthermore, there was a difference in the ability to support aerobic growth of *Fn* among the 12 cell lines. Some cell lines such as WIDR required less *Fn* (MOI of 0.001) to initiate successive *Fn* growth whereas SNU503 required *Fn* at MOI of 10 (Fig. 3a). The supernatants from co-cultures between WIDR and *Fn,* where *Fn* grew, induced γ-H2AX in various colon cancer cell lines (Fig. 3c, d), a hallmark of DNA double strand breaks (DSBs) and suggesting that *Fn* infection may be carcinogenic to infected tissues, whereas the supernatants from co-cultures between HCEC-1CT and *Fn,* where *Fn* growth was not permissive, did not induce γ-H2AX in the exposed colon cancer cell lines (Fig. 3e). Inclusion of the antibiotic metronidazole [13] in co-cultures between WIDR and *Fn* inhibited *Fn* growth (Fig. 3f) and abolished the supernatants’ ability to induce γ-H2AX in WIDR cells (Fig. 3g). Finally, the bacterial culture medium where *Fn* was anaerobically grown induced γ-H2AX in WIDR cells (Fig. 3h), indicating that *Fn* produces a factor that may cause DNA DSBs in mammalian cells.

Here we provide the initial report showing that heavy or moderate loads of *Fn* DNA are associated with MSI-H and L/E CRC, respectively. We have also identified evidence that *Fn* infection may cause DNA damage in infected colon tissues. It remains to be determined whether different degrees of *Fn* infection directly or indirectly impair DNA mismatch repair differently, resulting in MSI-H or L/E, or whether *Fn* opportunistically heavily infects MSI-H compared to L/E or MSS CRC. The host cell-dependent aerobic growth of *Fn* observed in this study may give further clues to answer these questions. However, our observation that *Fn* infection may trigger cellular DNA damage strongly suggests that *Fn* infection causes genetic and/or epigenetic alterations, initiating and/or promoting colorectal carcinogenesis. The identity and origin of the DNA damaging factor generated by *Fn* infection will need to be investigated.

## Supplementary information

**Additional file 1.** Additional materials and methods

## Data Availability

The datasets used and/or analyzed during the current study are available from the corresponding author by reasonable request.

## Abbreviations

*Fn*: *Fusobacterium nucleatum*
CRCs: Colorectal cancers
MSI-H: Microsatellite instability-high
CIMP: *CpG* island hyper-methylation phenotype
IAMA: Inflammation associated microsatellite alterations
MSI-L: Microsatellite instability-low
EMAST: Elevated level of microsatellite alterations at selected tetra-nucleotide repeats
L/E: MSI-L/EMAST
OR: Odd ratio
DSBs: Double strand breaks
MMR: Mismatch repair
CI: Confidential interval
MOI: Multiplicity of infection
NC: North Carolina
