# Supplementary material for: Fusobacterium nucleatum infection correlates with two types of microsatellite alterations in colorectal cancer and triggers DNA damage
Source: Gut Pathog. 2020 Sep 29;12:46. doi: 10.1186/s13099-020-00384-3 (PMC7526104; doi:10.1186/s13099-020-00384-3)
Supplement: Supplementary file 1 — Additional file 1. Additional materials and methods [file 13099_2020_384_MOESM1_ESM.docx]

***Fusobacterium nucleatum* Infection Correlates with Two Types of Microsatellite Alterations in Colorectal Cancer and Triggers DNA Damage**

Yoshiki Okita^1†^, Minoru Koi^1†^, Ryan Ross^2^, Bhramar Mukherjee^2^, Erika Koeppe^1^, Elena M. Stoffel^1^, Joseph Galanko^3^, Nikki McCoy^3,^ Temitope O Keku^3^, Yoshinaga Okugawa^4^, Takahito Kitajima^4^, Yuji Toiyama^4^, Eric Martens^5^, John M. Carethers^1,6*^

^1^Division of Gastroenterology and Hepatology, Department of Internal Medicine, University of Michigan, Ann Arbor, Michigan

^2^Department of Biostatistics School of Public Health, University of Michigan, Ann Arbor, Michigan

^3^Division of Gastroenterology and Hepatology, Departments of Medicine & Nutrition, University of North Carolina at Chapel Hill, Chapel Hill, North Carolina

^4^Department of Gastrointestinal and Pediatric surgery, Graduate School of Medicine Mie University, Mie, Japan

^5^Department of Microbiology and Immunology, University of Michigan, Ann Arbor, Michigan

^6^Department of Human Genetics and Rogel Cancer Center, University of Michigan, Ann Arbor, Michigan

*Corresponding author

^†^Contributed equally to this work

**SUPPLEMENTARY INFORMATION**

**Materials and Methods**

**CRC cohorts**

The discovery cohort consists of 91 unselected sporadic rectal cancer patients, and was obtained from the North Carolina Rectal Cancer Study cohort. An additional 213 unselected sporadic CRC patients that were obtained from the North Carolina site for The Cancer Care Outcomes Research and Surveillance consortium. The validation cohort consists of 174 unselected CRC patients obtained from Mie University, Japan. All institutions had IRB approval to conduct this study.

**Detection and quantification of *Fn* DNA from tumor tissues**

Tumor and normal tissues were microdissected separately from formalin-fixed paraffin-embedded (FFPE) sections (5 μm). Genomic DNA was isolated from microdissected tissues using a QIAamp DNA FFPE Tissue Kit (QIAGEN, Hilden, Germany). The standard curve method was used to determine absolute quantification of *Fn* DNA and tumor DNA. Genomic DNA from the *Fn* strain VPI4355 (ATCC, Manassas, VA) and genomic DNA from the human colon cancer cell line, DLD1, were used for DNA standard. Isolated DNA was quantified by Qubit Assay on a Qubit 4 fluorometer (Invitrogen, Waltham, MA). Quantification of *Fn* and human DNA was determined by quantitative PCR using SYBER Green master mix (Applied Biosystems, Foster City, CA). The PCR primer sets specific to *Fn* and to the human genome were designed to target the *nusG* gene of *Fn* [1] and the non-protein coding region of human chromosome 9p24 (chr9:224109-2241357; GRCH38/hg38). Each reaction contained 2.5 ng of DNA and assayed in duplicate in 10 ul reactions containing 1x final concentration of Power SYBER Green Master MIX (Applied Biosystems), 25uM of each primer in a 96-well optical PCR plate. Amplification and detection of DNA was carried out using the ABI 7900HT Sequence Detection System (Applied Biosystems). PCR reaction conditions were as follows: 2 min at 50C, 10 min at 95C and 40 cycles of 15 sec at 95C and 1 min at 54C. The primer sequences for each primer set were as follows: *Fn* forward primer, 5’-CTGGTGTCATTCTTCCAAAAATATCA-3’; Fn reverse primer, 5’-GATCAAGAAGGACAAGTTGCTG-3’; 9p24 forward primer, 5’-CTCTCCTCGATCCTCCCTTT-3’ and 9p24-reverse primer; 5’-AGGAAGCAACGAAACAATCG-3’. Reaction efficiencies for the Fusobacterium assay and the *9p24* assay were found to be 97% and 100%, respectively. *Fn* DNA load for each sample was determined by the following formula: *Fn* DNA load= absolute *Fn* DNA in picogram/absolute tumor genomic DNA in nanogram.

***Fn* and human cell cultures**

*Fn* EAVG_002 strain [2] was anaerobically grown in a chopped meat-based medium [3]. Bacteria were routinely grown at 37 °C in an anaerobic chamber (Coy Laboratory Products Inc, Grass Lake, Michigan). The human CRC cell lines, SNU503, DLD1, HCT116, RKO, LOVO, LS174T, HCA7, CACO2, SW620, SNU175, SNU407, WIDR, SNU81, HCEC-1CT, SW48 and NCI-H747, were grown in Dulbecco's modified Eagle medium with 10% fetal bovine serum.

**Detection and quantification of *Fn* DNA from tissue cultures**

One day before *Fn* infection, 10^4^ cells from each cell line were plated into the 96 well plate. The cells were infected with anaerobically grown *Fn* at MOI of 0.001, 0.01, 0.1, 1, 10 or 100. After 3 days of cultivation under 5% CO_2_/21% O_2_ conditions, a minimum number of MOI necessary for initiating and establishing *Fn* growth was determined for each cell line. *Fn* copy number present in the 3-day co-culture that received minimum amount of *Fn* for establishing *Fn* growth was also determined for each cell line. In separate experiments, 5X10^6^ WIDR cells were also infected with or without *Fn* EAVG_002 strain at MOI of 3 and cultivated with or without 30 uM of metronidazole for 5 days. Culture medium was collected from each culture and subjected to *Fn* copy number determination. The medium with Fn were heated at 95^o^C for 3 min, and used as a template for qPCR as described above. *Fn* copy number was calculated based on the estimated molecular size of 2.17 million base pairs [4].

**Western blotting**

Anti-γ-H2AX monoclonal mouse antibody (clone JBW301) was obtained from Millipore Sigma (Burlington, MA). Heat-denatured cell lysates (50 μg) were subjected to electrophoresis in a 10% polyacrylamide gel with sodium dodecyl sulfate. Separated proteins were electroblotted to a polyvinylidene difluoride membrane. The membrane was treated with primary antibody (1:2000) followed by treatment with horseradish peroxidase-conjugated goat anti-mouse immunoglobulin G. Signals were detected using Immobilon Western (Millipore) and captured by ImageQuant LAS4000 (General Electric, New York City, NY). Tubulin protein was used as reference marker for protein loading.

**Immunofluorescence microscopy**

Twenty-five thousand WIDR cells were seeded onto each well on 8-well chamber slides (Invitrogen) and incubated overnight. The cells were treated with filtered supernatants from WIDR cell cultures infected with *Fn*. After 9hr, the cells were fixed with 4% formaldehyde for 15 min followed by permeabilization with ice-cold methanol for 10 min. Slides were treated with 5% FBS/PBS (blocking/washing buffer) for 20 min before incubating with antibodies against γ-H2AX (1∶300) for 60 min at 37C. After washing with PBS, the slides were treated with secondary Alexa 488 conjugated anti-mouse antibody (1∶2000) for 60 min in dark. Slides were mounted with Prolong Gold mounting media with DAPI (Invitrogen). Pictures were taken using an Olympus DP72 Fluorescent microscope.

**Statistical Analysis**

The difference in *Fn* loads among MSI-H, L/E and MSS CRC was tested using the Wilcoxon rank test. The association between *Fn* infection and molecular subtypes and other variables including sex, age, tumor location and stage was tested using a logistic regression model with Firth bias correction. When p values were less than 0.05, the difference or association was labelled significant.

**References for Supplemental Information**

[1] Castellarin M, Warren RL, Freeman JD, Dreolini L, Krzywinski M, Strauss J, et al. Fusobacterium nucleatum infection is prevalent in human colorectal carcinoma. *Genome Res.* 2012 Feb;22(2):299-306.

[2] Dharmani P, Strauss J, Ambrose C, Allen-Vercoe E, Chadee K. Fusobacterium nucleatum infection of colonic cells stimulates MUC2 mucin and tumor necrosis factor alpha. *Infect Immun.* 2011;79(7):2597-2607.

[3] Hehemann JH, Kelly AG, Pudlo NA, Martens EC, Boraston AB. Bacteria of the human gut microbiome catabolize red seaweed glycans with carbohydrate-active enzyme updates from extrinsic microbes. *Proc Natl Acad Sci U S A.* 2012;109(48):19786-19791.

[4] Kapatral V, Anderson I, Ivanova N, Reznik G, Los T, Lykidis A, et al. Genome sequence and analysis of the oral bacterium Fusobacterium nucleatum strain ATCC 25586 *J Bacteriol.* 2002;184(7):2005-2018.
